# The performance of 11C-Methionine PET in the differential diagnosis of glioma recurrence

**DOI:** 10.18632/oncotarget.19024

**Published:** 2017-07-05

**Authors:** Weilin Xu, Liansheng Gao, Anwen Shao, Jingwei Zheng, Jianmin Zhang

**Affiliations:** ^1^ Department of Neurosurgery, Second Affiliated Hospital, School of Medicine, Zhejiang University, Hangzhou, Zhejiang, China; ^2^ Brain Research Institute, Zhejiang University, Hangzhou, Zhejiang, China; ^3^ Collaborative Innovation Center for Brain Science, Zhejiang University, Hangzhou, Zhejiang, China

**Keywords:** methionine, PET, glioma, recurrence, meta-analysis

## Abstract

Despite the advancement of neuroimaging techniques, it often remains a diagnostic challenge to distinguish recurrent glioma from lesions representing treatment effect. Preliminary reports suggest that 11C-methionine Positron emission tomography (PET) can assist in diagnosing true glioma recurrence. We present here a meta-analysis to assess the accuracy of 11C-methionine PET in identifying recurrent glioma in patients who had undergone prior therapy. A comprehensive search of the PubMed, Embase and Chinese Biomedical (CBM) databases yielded 23 eligible articles comprising 29 studies listed prior to November 20, 2016, representing 891 patients. In this report, we assess the methodological quality of each article individually and perform a meta-analysis to obtain the summary diagnostic accuracy of 11C-methionine PET in correctly identifying recurrent glioma. The pooled sensitivity and specificity are 0.88 (95% CI: 0.85, 0.91) and 0.85 (95% CI: 0.80, 0.89), respectively, with an area under the curve (AUC) for the summary receiver-operating characteristic curve (SROC) of 0.9352. We conclude that 11C-methionine PET has excellent diagnostic performance for differentiating glioma recurrence from treatment effect.

## INTRODUCTION

Gliomas are the most common type of primary malignant tumor found in the central nervous system, and patients suffering from high-grade glioma experience extremely high rates of morbidity and mortality [1]. Patients who have received comprehensive treatments that included surgery, chemotherapy and/or radiotherapy, often exhibit new lesions on contrast-enhanced CT or MRI during long-term follow-up. These traditional imaging modalities are largely insufficient to distinguish true tumor recurrence from other effects, namely pseudo-progression or radio-necrosis [2, 3]. Conventional or contrast-enhanced MRI was reported to own low specificity (24%) or low specificity (44.4%) in the differentiation of glioma from benign tissues [4, 5]. As the treatments for these conditions differ significantly, an accurate diagnosis of recurrent glioma is critical to minimize the likelihood of unnecessary and invasive treatments [6].

Positron emission tomography (PET) is a promising imaging modality that has been extensively applied in the field of neuro-oncology. The 11C-methionine has been reported to be an excellent tracer for PET imaging, and many prior studies have reported the use of 11C-methionine PET for identifying recurrent tumors [7–9]. In a previously published meta-analysis [10], the performance of 11C-methionine PET in distinguishing glioma recurrence was evaluated, but it included few studies and lacked a comprehensive subgroup analysis. Consequently, we present an enhanced meta-analysis to systematically evaluate the value of 11C-methionine PET for the identification of recurrent tumors.

## RESULTS

### Study screening and its characteristics

**Figure 1:**
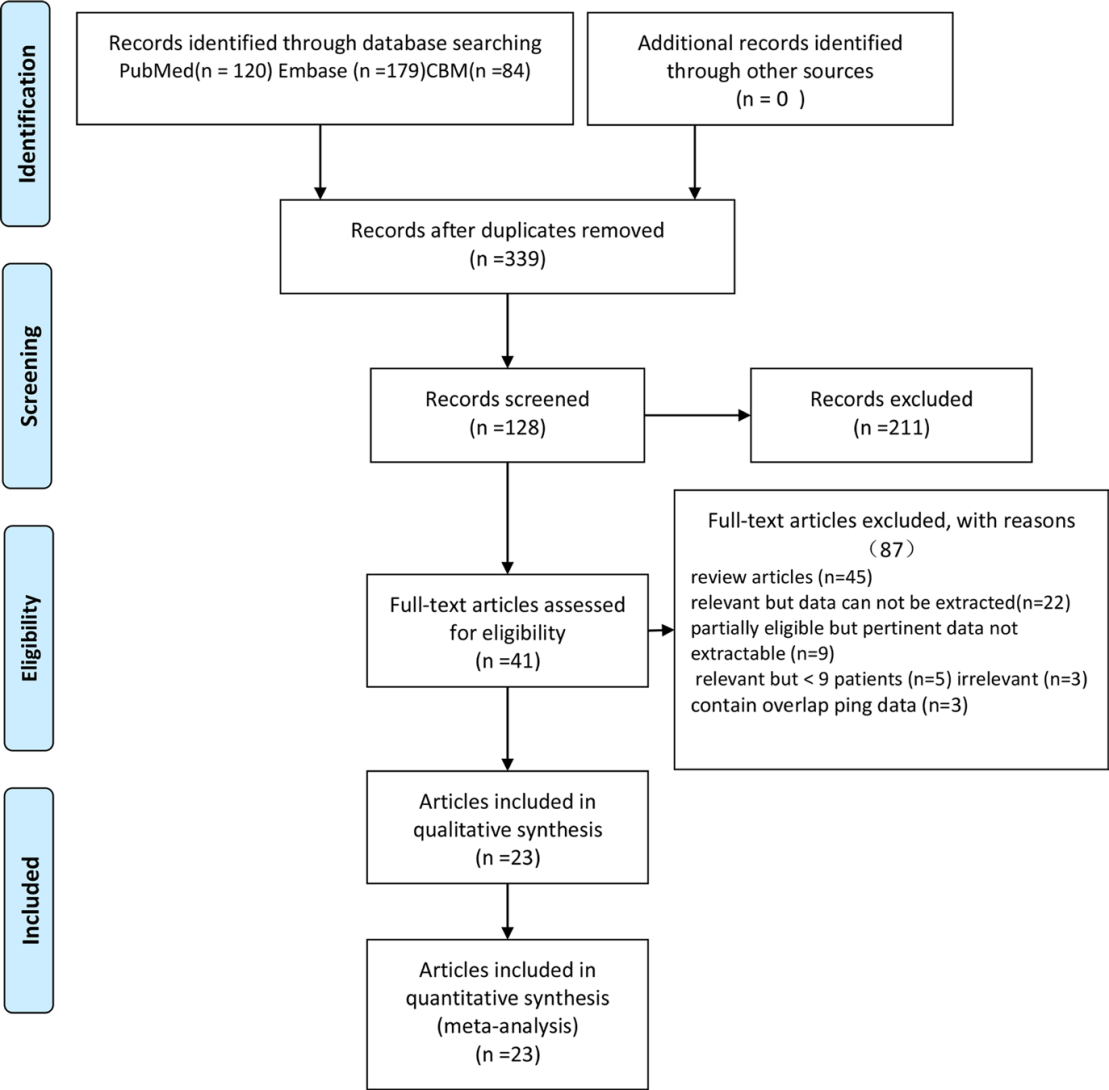
Flow diagram of the study selection process

Searches of pubmed, Embase and Chinese Biomedical databases (CBM) identified 120, 179 and 84 citations, respectively. After screening the records for duplications, only 41 of them were potentially eligible for further full text evaluation. In all, 23 articles with a total sample size of 891 patients representing 899 scans that met all inclusion and exclusion criteria were included for the meta-analysis [7–9, 11–30]. The PRISMA flow diagram of the study selection process is displayed in Figure 1, and the basic characteristics of all 23 articles are summarized in Table 1.

**Table 1:** Characteristics of studies included in the meta-analysis of 11C-methionine PET for the differential diagnosis of glioma recurrence

Author	Year	Country	Study design	cases	scans	Mean age,years (range)	M/F	Type of glioma	analysis method	Reference standard	Injected dose	Parameter	cutoff	tp	fp	fn	tn
Tae-Young Jung (a)	2016	Korea	R	42	42	45.6 (13-75)	23/19	HGG (42)	semi	his (12) or follow-up (30)	7 MBq/kg	T/Nmax	1.43	32	0	3	7
Tae-Young Jung (b)	2016	Korea	R	42	42	45.6 (13-75)	23/19	HGG (42)	semi	his (12) or follow-up (30)	7 MBq/kg	MTV	6.72	27	0	8	7
Rajnish Sharma	2016	India	R	64	64	5-56	41/23	HGG (26);LGG (38)	semi	his (12) or follow-up (52)	370 MBq	T/Nmax	1.47	45	2	0	#
Yuzo Terakawa	2016	Japan	R	26	32	NA	NA	HGG (20);LGG (6)	semi	his (22) or follow-up (10)	6 MBq/kg	T/Nmean	1.58	12	4	4	#
yan wenming	2016	China	P	35	35	42.4 (31-72)	21/14	HGG (22);LGG (13)	semi	his (35)	450 MBq	T/Nmean	NA	23	0	2	#
J.R. Garcia (a)	2016	Spain	NA	30	30	55	16/14	HGG (30)	visual	his (3) or follow-up (27)	6 MBq/kg	visual	NA	21	2	0	7
J.R. Garcia (b)	2016	Spain	NA	30	30	55	16/14	HGG (30)	semi	his (3) or follow-up (27)	6 MBq/kg	Tmax/Nmean	2.35	19	0	2	9
Ryogo MiNAmimoto1 (a)	2015	Japan	R	31	31	NA	NA	HGG (31)	visual	his or follow-up (NA)	418.7 MBq	NA	NA	18	5	3	5
Ryogo MiNAmimoto1 (b)	2015	Japan	R	31	31	NA	NA	HGG (31)	semi	his or follow-up (NA)	418.7 MBq	SUVmax	1.4	14	4	7	6
Ryogo MiNAmimoto1 (c)	2015	Japan	R	31	31	NA	NA	HGG (31)	semi	his or follow-up (NA)	418.7 MBq	SUVmean	1.2	11	1	10	9
Maria M	2014	India	P	29	29	NA	20/9	HGG (29)	semi	his (22) or follow-up (7)	7 MBq/kg	T/Nmean	1.58	18	2	1	8
Shunshke TAKENAKA	2014	Japan	R	50	50	45.56	26/24	HGG (50)	semi	his (50)	7 MBq/kg	T/Nmean	2.51	31	2	3	#
T.YU.SKVORTSOVA	2014	Russia	P	72	72	36 (3-68)	35/37	HGG (51);LGG (21)	semi	his (17) or follow-up (55)	NA	T/Nmean	1.9	25	1	5	#
yu lei	2013	China	P	22	22	42 (28-70)	12/10	HGG (13);LGG (9)	visual	his (22)	555-740 MBq	NA	NA	16	0	1	5
Hajime Shishido (a)	2012	Japan	R	21	21	54 (22-71)	11/10	HGG (21)	semi	his (13) or follow-up (8)	215 MBq	Tmax/Nmean	2.69	12	1	3	5
Hajime Shishido (b)	2012	Japan	R	21	21	54 (22-71)	11/10	HGG (21)	visual	his (13) or follow-up (8)	215MBq	NA	NA	15	5	0	1
Madhavi Tripathi (a)	2012	India	P	35	35	33.7 (5-65)	23/12	HGG (20);LGG (15)	semi	his (14) or follow-up (21)	550-740 MBq	T/Nmax	1.9	23	1	1	#
Madhavi Tripathi (b)	2012	India	P	35	35	33.7 (5-65)	23/12	HGG (20);LGG (15)	visual	his (14) or follow-up (21)	550-740 MBq	NA	NA	24	0	0	#
Yunqin Liu	2011	China	P	30	30	41 (11-69)	21/9	HGG (11);LGG (19)	semi	his (19) or follow-up (11)	740 MBq	T/Nmean	1.58	18	1	0	#
Dongli LI	2011	China	R	46	46	37.5 (7-69)	34/12	HGG (32);LGG (14)	visual	his (22) or follow-up (24)	370-550MBq	NA	NA	34	1	2	9
Yong Hwy Kim	2010	Korea	R	10	10	46.1	8/2	HGG (10)	semi	his (3) or follow-up (7)	NA	T/Nmax	2.64	3	0	1	6
ANCA-LIGIA GROSU	2010	Norway	P	29	29	NA	NA	HGG (25);LGG (4)	semi	his (17) or follow-up (12)	185-370MBq	T/Nmean	1.5	27	0	2	0
Chunlin Ye	2009	China	R	28	28	47.3 (43-71)	21/7	LGG (28)	semi	his (15) or follow-up (13)	5.5-7.4 MBq/kg	SUVmax	2.5	19	1	1	7
Takeshi NAkajima	2009	Japan	R	18	18	45 (14-67)	12/6	HGG (18)	semi	his (14) or follow-up (4)	200-550 MBq	T/Nmean	2	6	0	1	#
Shiquan Wang	2005	China	NA	22	22	36 (9-76)	NA	NA	visual	his or follow-up (NA)	370-555MBq	NA	NA	18	0	0	4
NAohiro TSUYUGUCHI	2004	Japan	R	11	11	35.5 (23-62)	8/3	HGG (11)	visual	his (6) or follow-up (5)	370MBq	NA	NA	6	2	0	3
Koen Van Laere1	2004	Belgium	R	30	30	40.4	21/9	HGG (17);LGG (13)	semi	his (5) or follow-up (25)	220MBq	T/Nmean	2.2	13	3	5	9
Yukihiko SONODA	1998	Japan	R	10	12	41.5 (21-68)	6/4	HGG (5);LGG (5)	visual	his (6) or follow-up (6)	NA	NA	NA	5	0	1	6
T. OGAWA	1991	Japan	R	10	10	41.7 (2-60)	3/7	HGG (7);LGG (3)	visual	his (9) or follow-up (1)	500-1480 MBq	NA	NA	7	0	0	3

F = female, FN = false negative, FP = false positive, his=histology, HGG = high grade glioma, LGG = low grade glioma, M = male, MTV = metabolic tumor volume, NA = not available, P = prospective, R = retrospective, Semi = semi-quantitative, SUV= standardized uptake values, PET = positron emission tomography, T/N=tumor/normal, TN = true negative, TP = true positive.

The final analysis included 23 articles covered 29 studies, of which 18 were retrospective and 8 were prospective. 3 studies did not mention the study design. The sample sizes ranged from 10 to 72. The most frequent parameter, the ratio of mean uptake of tumor to normal background (T/Nmean), was employed in nine studies. The next most frequent parameter was the ratio of maximum uptake of tumor to normal background (T/Nmax), which was used in four studies. There were likewise two studies that utilized either the tumor max/normal mean or standardized uptake value (SUVmax). Lastly, one study compared a novel parameter, metabolic tumor volume (MTV), with conventional T/Nmax. Image analysis was performed visually in 10 studies and semi-quantitatively in 19 studies. Twenty articles included both histopathology and clinical follow-up, while three articles solely tracked pathology.

**Figure 2:**
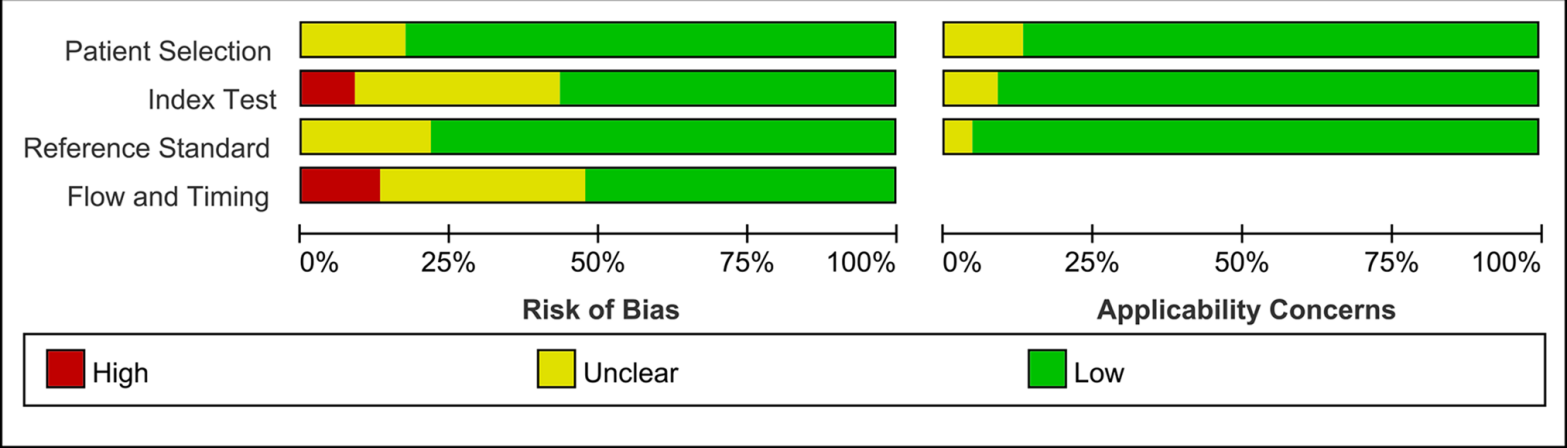
Methodological quality analysis of the 23 eligible articles using QUADAS-2 tool

We qualitatively judged the quality test of each study and the summary analysis is shown in Figure 2. We found that most of the studies had low or indeterminate risk of bias.

### Quantitative analysis

**Figure 3:**
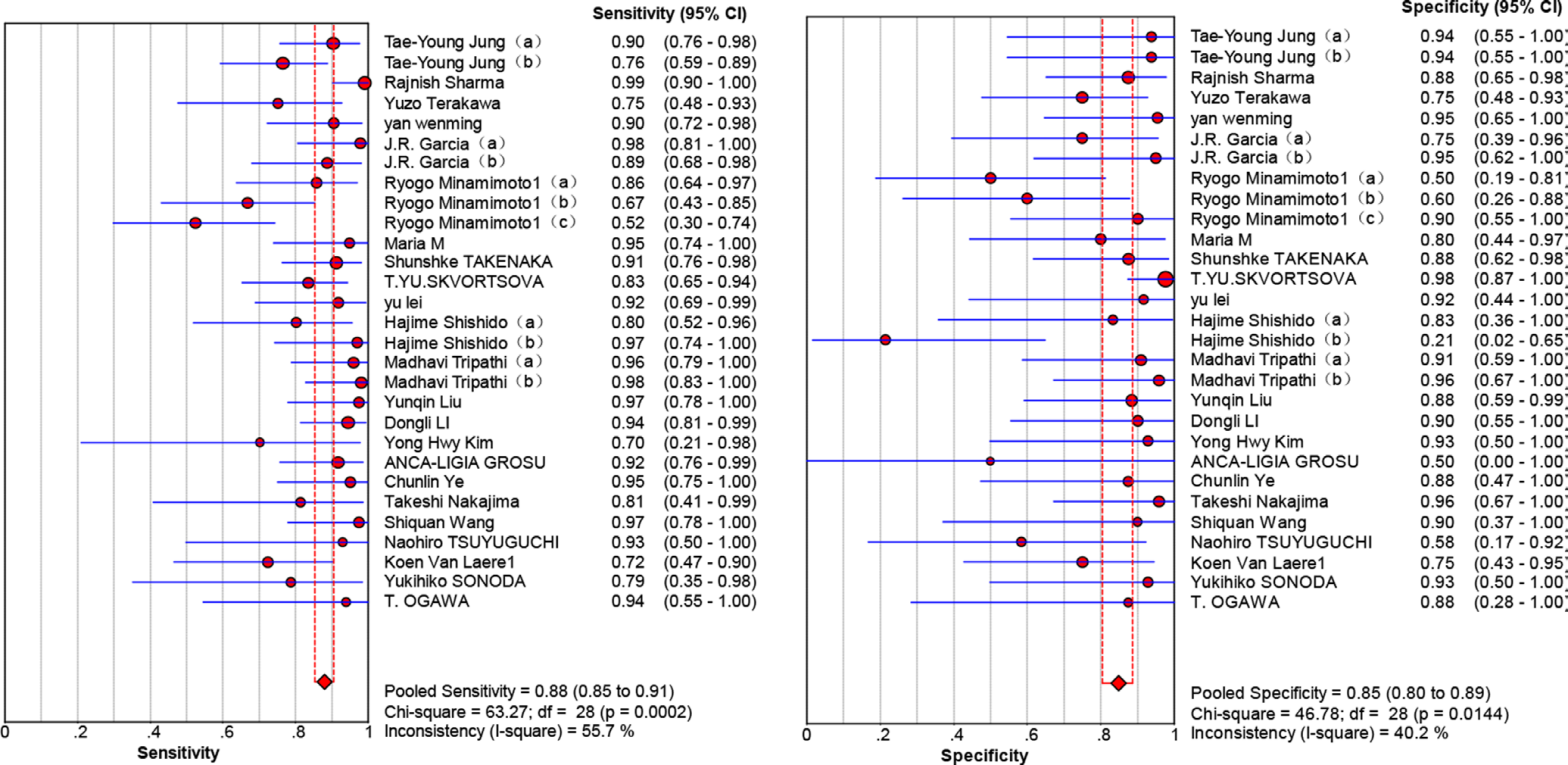
Forest plot showing the sensitivity and specificity for the differentiation of glioma recurrence

**Figure 4:**
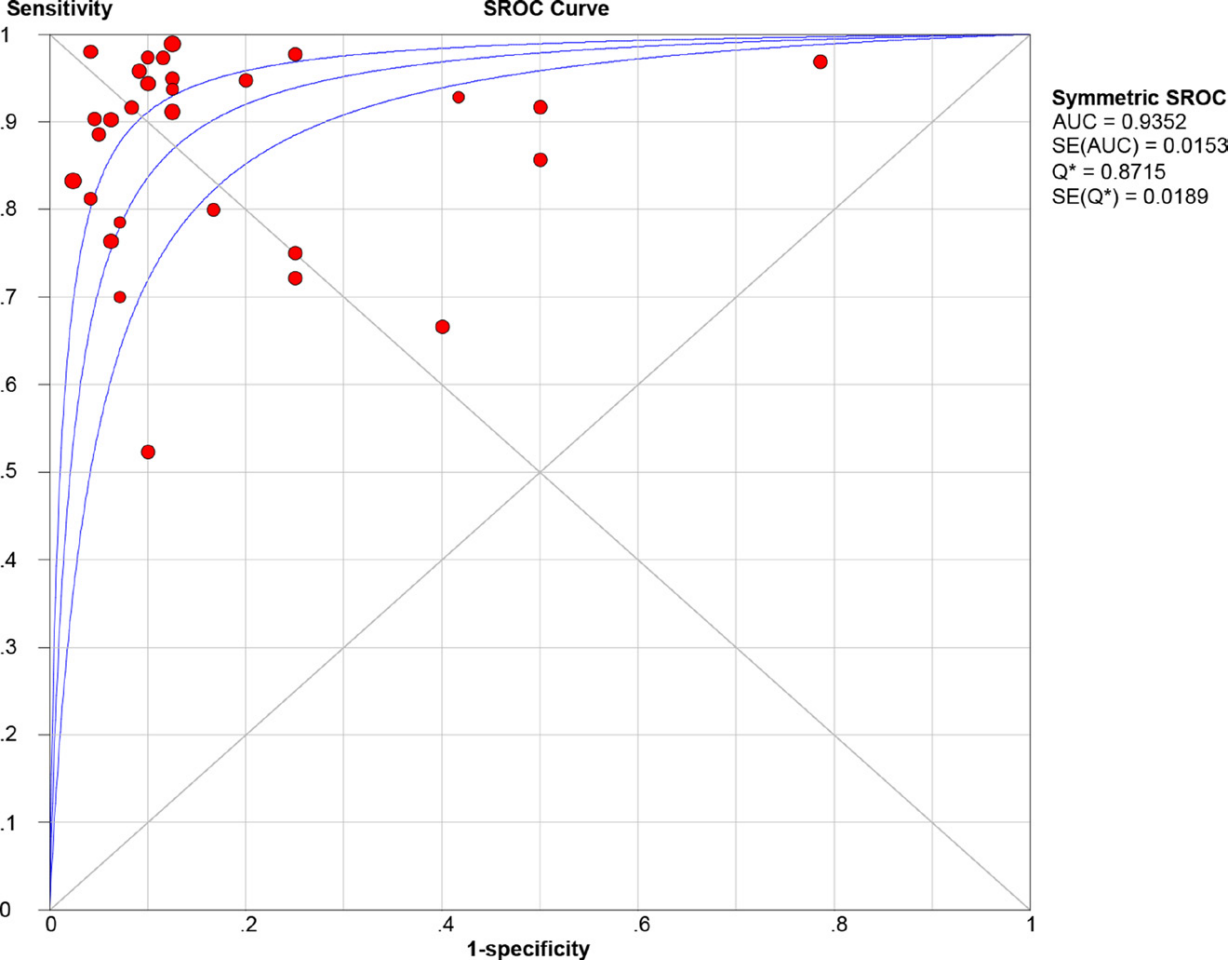
Summary receiver-operating characteristic curve (SROC)

Twenty-nine studies that represented 899 11C-methionine PET scans were analyzed to identify recurrent tumors after undergoing primary surgery and adjuvant therapy. The threshold effect was initially tested and the Spearman correlation coefficient for the included studies was 0.096 (*P* = 0.621), revealing no discernible threshold effect. There was no significant evidence of heterogeneity (I2 = 34.7%) observed in the overall analysis, so the fixed-effects coefficient binary regression model was chosen. Among the included studies, SEN has a scope from 0.52 to 1, with all but two greater than 0.7, while the SPE displayed a wider scope from 0.21 to 1. The summary weighted values of overall analysis are as follows: SEN: 0.88 (95% CI: 0.85, 0.91); SPE: 0.85 (95% CI: 0.80, 0.89); LR+: 5.35 (95% CI: 3.29, 8.70); LR−: 0.16 (95% CI: 0.11, 0.23); DOR: 35.30 (95% CI: 22.91, 54.39). Forest plots for the 29 included studies displayed in Figure 3, and the AUC under the SROC was 0.9352 (Figure 4).

Twelve studies utilized histology as the reference standard. The pooled SEN and SPE were: 0.92 (95% CI: 0.87, 0.95) and 0.81 (95% CI: 0.70, 0.90), respectively, corresponding to summary LR+ of 4.62 (95% CI: 2.76, 7.73) and LR- of 0.12 (95% CI: 0.07, 0.20). The summary DOR was 44.28 (95% CI: 18.23, 107.5) and the AUC under the SROC was 0.9494. Nine other studies utilized follow-up as the reference standard. The summary SEN and SPE: 0.79 (95%CI: 0.62, 0.91) and 0.86 (95% CI: 0.73, 0.94), respectively, corresponding to summary LR+ of 3.86 (95% CI: 1.8, 8.28) and LR- of 0.36 (95% CI: 0.19, 0.69). The summary DOR was 12.89 (95% CI: 3.6, 46.24), and the AUC under the SROC was 0.8595. The AUC analysis demonstrated no statistically significant difference between these two parameters (P_interaction_ = 0.184).

There were 4 studies that used T/Nmax (range: 1.43, 2.64) as a parameter. The summary SEN and SPE were: 0.94 (95%CI: 0.88, 0.98) and 0.90 (95% CI: 0.78, 0.97), respectively, corresponding to summary LR+ of 9.51 (95% CI: 3.9, 23.22) and LR- of 0.09 (95% CI: 0.03, 0.31). The summary DOR was 174.06 (95% CI: 37.37, 810.64), and the AUC under the SROC was 0.97. Nine other studies adopted T/Nmean (range: 1.50, 2.51) as parameter. The summary SEN and SPE were: 0.88 (95%CI: 0.82, 0.92) and 0.89 (95% CI: 0.82, 0.94), respectively, corresponding to summary LR+ of 6.94 (95% CI: 4.35, 11.1) and LR- of 0.16 (95% CI: 0.11, 0.24). The summary DOR was 38.59 (95% CI: 19.17, 77.69) and the AUC for the SROC was 0.934. The AUC analysis demonstrated no statistically significant difference between these two parameters (P interaction = 0.849).

Regarding the analysis method, 10 studies utilized visual assessment. The summary SEN and SPE were: 0.94 (95%CI: 0.90, 0.97) and 0.76 (95% CI: 0.64, 0.85), respectively, corresponding to summary LR+ of 4.08 (95% CI: 1.72, 9.70) and LR- of 0.09 (95% CI: 0.05, 0.16). The summary DOR was 40.84 (95% CI: 17.54, 95.08), with an AUC for the SROC of 0.9632. And 19 studies were semi-quantitative. The summary SEN and SPE: 0.86 (95%CI: 0.82, 0.89) and 0.88 (95% CI: 0.83, 0.92), respectively, corresponding to a summary LR+ of 6.78 (95% CI:4.76, 9.65) and LR- of 0.18 (95% CI: 0.12, 0.28). The summary DOR was 33.68 (95% CI: 20.34, 55.75), and the AUC for the SROC was 0.9338. Analysis of the AUC again failed to show a statistically significant difference between these two subgroups (Pinteraction = 0.006 < 0.05). The results of subgroup analyses by study design (prospective versus retrospective) and grades (HGG versus LGG) are shown in Table 2.

**Table 2:** Subgroup analyses of diagnostic accuracy variables

Category	studies, *n*	Scans, *n*	Threshold effects, *p* value	I^2^	SEN (95% CI)	SPE (95%CI)	LR+(95% CI)	LR-(95% CI)	DOR (95% CI)	AUC(SE)	P_interaction_
**Overall**	29	899	0.621	34.70%	0.88 (0.85–0.91)	0.85 (0.80–0.89)	5.35 (3.29–8.70)	0.16 (.011–0.23)	35.30 (22.91–54.39)	0.9352 (0.0153)	
**Parameters**											0.849
T/Nmax	4	151	0.2	0%	0.94 (0.88–0.98)	0.9 (0.78–0.97)	9.51 (3.9–23.22)	0.09 0.03–0.31)	174.06 (37.37–810.64)	0.97 (0.187)	
T/Nmean	9	325	0.983	37.10%	0.88 (0.82–0.92)	0.89 (0.82–0.94)	6.94 (4.35–11.1)	0.16 (0.11–0.24)	38.59 (19.17–77.69)	0.934 (0.0253)	
**References**											0.184
His	12	228	0.283	0%	0.92 (0.87–0.95)	0.81 (0.70–0.90)	4.62 (2,76–7.73)	0.12 (0.07–0.20)	44.28 (18.23–107.5)	0.9494 (0.0212)	
Follow-up	9	67	0.225	0%	0.79 (0.62–0.91)	0.86 (0.73–0.94)	3.86 (1.8–8.28)	0.36 (0.19–0.69)	12.89 (3.6–46.24)	0.8595 (0.0643)	
Grade											0.942
HGG	19	475	0.071	2.30%	0.85 (0.81–0.89)	0.80 (0.73–0.86)	4.03 (2.36–6.87)	0.19 (0.15–0.25)	24.64 (14.18–42.81)	0.9131 (0.0221)	
LGG	6	71	0.086	0%	0.89 (0.77–0.96)	0.80 (0.59–0.93)	4.54 (2.03–10.17)	0.13 (0.06–0.3)	26.66 (7.4–96.07)	0.9181 (0.0651)	
**Design**											0.006
Prospective	8	287	0.568	0%	0.92 (0.88–0.96)	0.93 (0.86–0.97)	11.44 (5.89–22.20)	0.09 (0.05–0.16)	168.35 (61.26–462.61)	0.9757 (0.0106)	
Retrospective	18	530	0.523	31.70%	0.85 (0.81–0.88)	0.80 (0.73–0.86)	4.06 (2.35–7.02)	0.22 (0.15–0.33)	21.01 (12.83–34.41)	0.9013 (0.0252)	
**Analysis methods**											0.311
Visual	10	240	0.854	22.60%	0.94 (0.90–0.97)	0.76 (0.64–0.85)	4.08 (1.72–9.70)	0.090 (0.05–0.16)	40.84 (17.54–95.08)	0.9632 (0.0222)	
Semi	19	659	0.943	42%	0.86 (0.82–0.89)	0.88 (0.83–0.92)	6.78 (4.76–9.65)	0.18 (0.12–0.28)	33.68 (20.34–55.75)	0.9338 (0.0187)	

FN = false negative, FP = false positive, his=histology, HGG = high grade glioma, LGG = low grade glioma, Semi = semi-quantitative, T/N=tumor/normal, SEN=sensitivity, SPE=specificity, LR+=positive likelihood ratio, LR-=negative likelihood ratio, DOR=Diagnostic Odds Ratio, AUC=area under curve.

Some other subgroups were ineligible for the subgroup meta-analysis because of data limitations.

### Heterogeneity analysis

No severe heterogeneity was noted in the summary analysis (I^2^ = 34.7%).

### Publication bias

**Figure 5:**
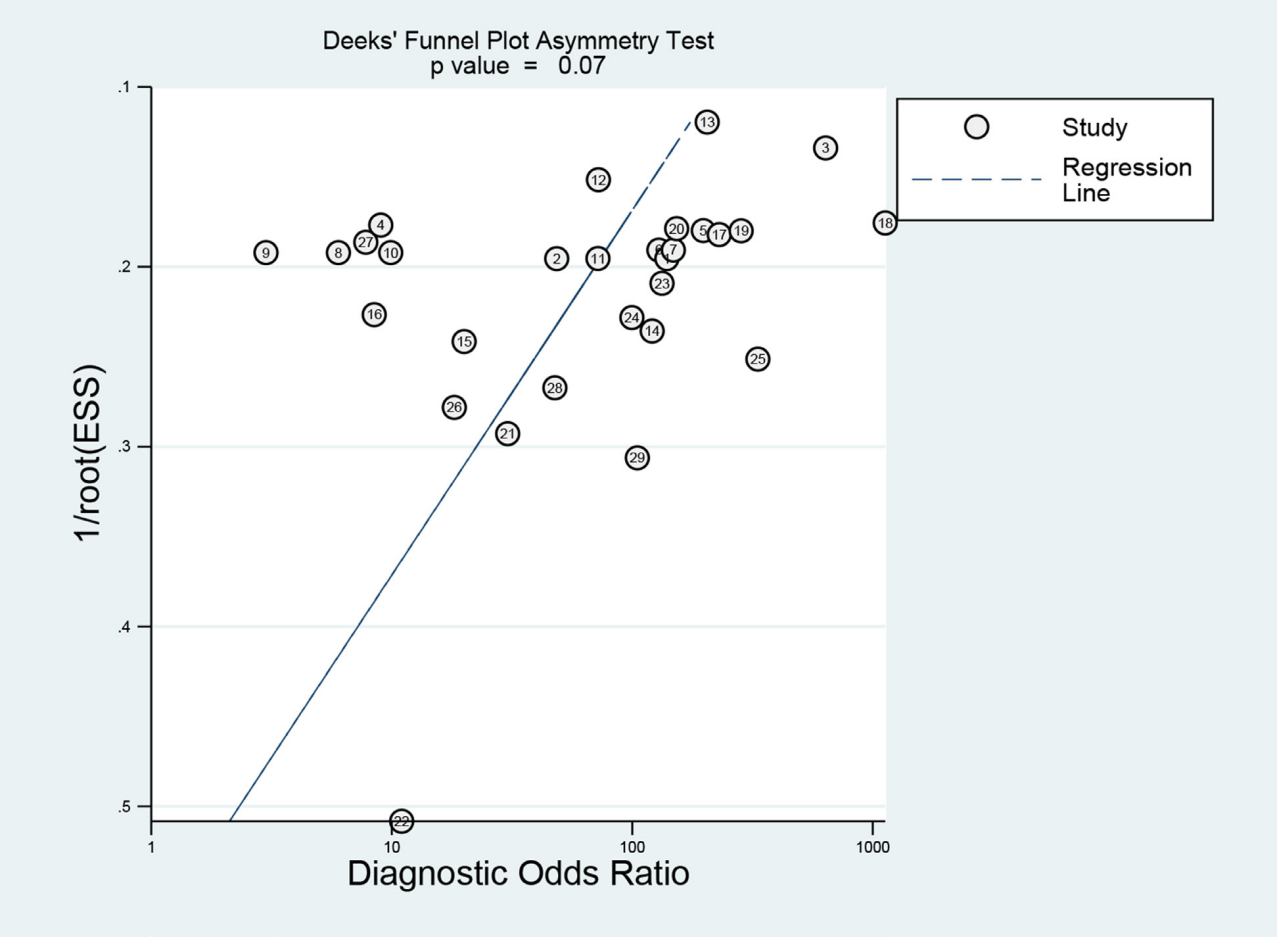
Funnel plot of publication bias

Deek’s funnel plot asymmetry test indicated no evidence of publication bias across the included studies (*p* = 0.07) (Figure 5).

## DISCUSSION

PET is a promising imaging modality in the differential diagnosis of brain tumors. The most frequently tracer been studied was FDG. 11C-methionine is a novel amino tracer which is extensively clinically utilized as it accumulates in the rapidly proliferating tumors. Many studies have demonstrated the superiority of 11C-methionine over FDG [31, 32]. So, we perform a meta-analysis to test the value of 11C-methionine PET in identifying true glioma recurrence.

This meta-analysis, which includes 29 studies that represent 899 scans, summarizes the diagnostic performance of 11C-methionine PET in positively identifying glioma recurrence in patients where it was suspected by conventional imaging modalities, namely as CT or MRI. Results of quantitative analysis indicated that 11C-methionine PET had a high level of diagnostic accuracy (AUC = 0.9352), independent of glioma grade, study design or reference standard. Furthermore, the DOR incorporated diagnostic efficacy of the sensitivity and specificity data into a single value [32]. The summary DOR for the overall analysis was 35.3, which also showed the utility of 11C-methionine PET in distinguishing glioma recurrence from other lesions. There does not appear to be heterogeneity or publication bias in the overall analysis, suggesting statistical reliability of these results. The new imaging changes differ on the conventional MRI images after chemoradiotherapy, such as necrosis, pseudoprogression, and vascular events like stroke. This study mainly focused on the performance of 11C-methionine PET in the differentiation of glioma recurrence from necrosis or pseudoprogression. Kawai N, et al. reported that intracerebral hemorrhages and cerebral infarctions demonstrated mild to moderate MET uptake while tumor tissues showed high MET uptake [33].

Two distinct reference standards, histopathology and clinical follow-up, were used to assess for recurrence of tumor. However, the adoption of inconsistent reference standards potentially overrated the diagnostic accuracy of the test [34, 35]. Thus, subgroup analysis was conducted and there was no significant difference in the diagnostic performance of those two standards (Pinteraction=0.184). Conventionally, histopathological diagnosis was adopted as the gold standard in the identification of recurrence. However, the histopathologic findings could not be obtained from every patient due to ethical issues. Surgery or biopsy, which would increase the risk of morbidity and mortality, was not done for patients with no evidence of glioma recurrence or progression. Thus, the use of serial follow-up to evaluate for subsequent recurrence is an acceptable alternative to tissue acquisition in those cases.

Only two parameters, namely T/Nmean and T/Nmax, met all the criteria for subgroup analysis. For T/Nmean group, the AUC (0.934) indicated a high diagnostic accuracy in identifying glioma recurrence. In contrast, T/Nmax seemed to have a higher level of diagnostic accuracy with AUC under the SROC of 0.970. However, likelihood ratios are more meaningful when used in the evaluation of diagnostic accuracy of a test. LR > 10 or < 0.1 indicates a high level of accuracy [35]. For T/Nmax subgroup, the LR+ was 9.51, which indicates that glioma recurrence is nearly 10 times more likely to be positive in the PET scan than that of the benign lesion. Conversely, a LR− of 0.09 indicates that if value of the parameter was lower than the relevant cut-off value, the chance of glioma recurrence would be 9%. Nevertheless, the comparison of the AUC between these two parameters shown no significant difference (Pinteraction=0.849). More studies would be required to incorporate other parameters into comparison in order to select an optimal test.

There were 10 studies that utilized visual assessment, with 19 others adopting a semi-quantitative method in image analysis. AUCs for the SROC of these two methods were 0.9632 and 0.9338, respectively, implying that they both had a high level of diagnostic accuracy. Although this implies that the visual assessment had a more accurate diagnostic performance than the semi-quantitative method, the difference of AUC between these two methods had no statistical significance (Pinteraction=0.311). The results of visual assessment varied due to different criteria and interpreters, so this likely introduced some bias into our pooled data. The same dilemma existed with semi-quantitative assessment due to different application of parameters and the setting of corresponding cutoff values. Therefore, we should be cautious when trying to interpret these results. Further evaluation and standardization of techniques should be attended before it is widely clinically used.

### Limitations

However, some limitations in our meta-analysis should be mentioned, despite the 11C-methionine PET displaying a high diagnostic accuracy.

First, the included studies varied greatly in study design, pathology grading, image analysis, parameter thresholds, sample sizes and patient selection. This could potentially increase the clinical heterogeneity.

Second, although 29 studies with 899 total scans were included, there were limited data for the subgroup analysis of different parameters, such as SUVmax, SUVmean or MTV. More studies utilizing other parameters in the comparison are needed to select an optimal parameter for the test.

Finally, as only English and Chinese publications with full text were included in this meta-analysis, that may leave out some eligible studies that were either unpublished or reported in other languages.

In summary, our meta-analysis indicates that 11C-methionine PET had a high level of diagnostic accuracy for identifying recurrent tumors. However, the variability of parameter, optimal thresholds, and other characteristics from the included studies suggests that further evaluation and standardization is needed before relying exclusively on 11C-methinoine PET imaging to diagnose recurrent glioma.

## MATERIALS AND METHODS

### Search strategy

The PubMed, Embase and Chinese Biomedical databases were searched comprehensively to select eligible published articles (up to 20 Nov 2016). The following key words were used for each topic: (a) “glioma” or “gliomas” or “brain neoplasm” or “astrocytoma” or “oligdendrocytoma” or “ependymoma” or “oligodendroglioma”, (b) “PET” or “positron emission tomography”, (c) “recurrent” or “recurrence” or “relapse” or “regrowth”, and (d) “11C-methionine” or “methionine” or “L-11C-MET” or “11C-MET” or “carbon-11 methionine”. Furthermore, the reference lists of all eligible studies were hand-searched for any remaining relevant articles.

### Inclusion and exclusion criteria

The following Inclusion criteria were used: (1) 11C-methionine PET was used to distinguish glioma recurrence from any other benign lesions after chemoradiotherapy (necrosis, pseudoprogression), (2) the pathology of the tumors was proven histologically, (3) the ‘gold standard’ was histopathology and/or clinical follow-up. (4) the study had at least 10 patients, (5) the sensitivity and specificity could be calculated from the data, (6) there were no overlapping subjects across publications, and (7) the language of publication was either Chinese or English. The following types of studies were excluded: reviews, letters, editorials, abstracts, case reports, proceedings, and personal communications.

Data from the potentially eligible studies were extracted and summarized individually by two of the reviewers (Weilin Xu and Liansheng Gao). Any disagreement was settled by a third reviewer (J.M.Zhang).

### Data extraction and quality assessment

Final data from the eligible studies were extracted and assessed individually by two of the reviewers (Weilin Xu and Anwen Shao). The following basal characteristics were obtained: authors, years, country, study design, age and gender, number of patients included in each study, scans, type of glioma, pathology, reference standard, analysis method, parameters and its cut-off value. The numbers of true positive (TP), false positive (FP), false negative (FN) and true negative (TN) from each study were calculated. And we use the Quality Assessment Tool for Diagnostic Accuracy Studies version 2 (QUADAS-2) to evaluate the methodological quality for each included article [36]. Any discrepancies were resolved by the adjudicating senior reviewer.

### Statistical analysis

First, the threshold effect was determined by calculating the Spearman correlation coefficient between the logit of SEN and the logit of (1−SPE). A threshold effect was if the observed correlation had a *p* value < 0.05.

Second, we assessed the level of heterogeneity among studies by calculating the chi-squared test and the inconsistency index (I2) of the diagnostic odds ratio (DOR). We concluded that Significant heterogeneity existed if the computed *p* value was less than 0.05 or I2 was greater than 50%. A random-effects coefficient binary regression model was applied if significant heterogeneities were observed and a meta-regression analysis was then carried out to explore the potential source of heterogeneity. Otherwise, we adopted the fixed-effects coefficient binary regression model [37, 38].

Third, pooled analysis for the sensitivity, specificity, likelihood (LRs) and diagnostic odds ratios (DOR) for 11C-methionine PET with corresponding 95% confidence intervals (CIs) was performed as the primary meta-analysis. The same principle was applied to our subgroup analysis. We added a value of 0.5 to all cells of studies that had SENs or SPEs of 100%.

Next, receiver-operating characteristic curve (SROC) was constructed, and the area under the curve (AUC) and Q* index (it was referred to the point on the SROC at which SEN and SPE are equal) was computed. The guidelines to interpret the value of AUC were as follows: low accuracy, 51% to 70%; moderate accuracy, 71% to 90%; high accuracy, > 90% [39].

To investigate the heterogeneity further, these studies were classified into some subgroups which contain homogeneous characteristics of studies according to similar parameter. Each subgroup constructed should contain at least three studies. The Z test was used to do the comparisons among subgroups, which suggested a significant difference among subgroups if *p* < 0.05. The statistical analyses mentioned above were conducted with Meta-Disc statistical software version 1.4 [40].

Finally, publication bias was evaluated by Deek’s funnel plot and linear regression method [41] using a value of *p* < 0.05 in the linear regression model to indicate significant asymmetry [32].

This statistical analysis was performed using Stata statistical software 13.0 (StataCorp LP, College Station, TX).

## Abbreviations

AUC: area under the curve
CBM: Chinese Biomedical
DOR: Diagnostic Odds Ratio
CI: confidence intervals
F: female
FN: false negative
FP: false positive
HGG: high grade glioma
LGG: low grade glioma
LR+: positive likelihood ratio
LR-: negative likelihood ratio
M: male
MTV: metabolic tumor volume
NA: not available
P: prospective
PET: Positron Emission Tomography
R: retrospective
Semi: semi-quantitative
SROC: summary receiver operating characteristic curve
SEN: sensitivity
SPE: specificity
Semi: semi-quantitative
SUV: standardized uptake values
T/N: tumor/normal
